# Evaluating the efficacy and microenvironment changes of HER2 + gastric cancer during HLX02 and Endostar treatment using quantitative MRI

**DOI:** 10.1186/s12885-022-10136-y

**Published:** 2022-10-03

**Authors:** Jianye Liang, Wei Dai, Zhipeng Li, Xiangjing Liang, Mingjia Xiao, Chuanmiao Xie, Xinming Li

**Affiliations:** 1 Department of Medical Imaging, State Key Laboratory of Oncology in South China, Collaborative Innovation Center for Cancer Medicine, Sun Yat-Sen University Cancer Center, Guangzhou, China; 2 Ultrasound Medical Center, Zhujiang Hospital, Southern Medical University, Guangzhou, China; 3 Department of Hepatobiliary Surgery II, Zhujiang Hospital, Southern Medical University, Guangzhou, China; 4 Department of Radiology, Zhujiang Hospital, Southern Medical University, Guangzhou, China

**Keywords:** HLX02, IVIM-DWI, Gastric cancer, HER2, Endostar, Vascular normalization

## Abstract

**Background and objectives:**

Trastuzumab is an important targeted drug for HER2-positive gastric cancer. The treatment efficacy of a more cost-effective and accessible trastuzumab biosimilar, HLX02, was not well investigated, especially when combined with antiangiogenic treatment. In addition, the tumour microenvironment detected by functional MRI was still unclear during treatment. This study attempts to evaluate the therapeutic effect of antiangiogenic agents combined with HLX02 in a HER2-positive gastric cancer xenograft model and to detect microenvironmental changes using intravoxel incoherent motion diffusion-weighted imaging (IVIM-DWI).

**Materials and methods:**

We subcutaneously injected MKN-45 human gastric cancer cells into BALB/C nude mice to establish a tumour model. Twenty-eight mice were divided into four groups and treated with saline (Group 1), Endostar (Group 2), trastuzumab biosimilar HLX02 (Group 3), or the combination of Endostar and HLX02 (Group 4). We then performed IVIM-DWI before and at different time points after treatment. HE, HER2, TUNEL, E-cadherin staining, and α-SMA and CD31 double-staining were used to confirm the pathological changes.

**Results:**

Group 4 demonstrated the smallest tumour volume at the end of treatment. The D value in Group 4 increased more dramatically, with the highest value on Day 20, compared with the other groups. Perfusion-related parameters (D* and f values) in Groups 2 and 4 increased initially and reversed after Day 10. Group 4 showed the lowest CD31 and HER2 and the highest TUNEL- and E-cadherin-positive staining rates. The D value was positively correlated with TUNEL but negatively correlated with HER2 staining. The D* and f values had positive correlations with CD31 and E-cadherin expression and the vessel maturity index.

**Conclusions:**

The trastuzumab biosimilar drug HLX02 exhibited good treatment efficacy in HER2-positive gastric cancer, especially when combined with Endostar. IVIM-DWI can noninvasively monitor the process of vascular normalization and reflect the treatment effect early at the molecular level.

## Introduction

Gastric cancer remains a highly concerning cancer worldwide, ranking fifth in incidence and fourth in mortality in 2020 [1]. Systemic chemotherapy is the first-line option for patients with advanced, unresectable disease. However, the prognosis remains poor due to the low targeting ability of chemotherapy and the advanced stage at the time of diagnosis. Human epidermal growth Factor 2 (HER2) is an important factor mediating cell proliferation, differentiation and survival [2]. It is overexpressed not only in breast cancer but also in gastric cancer [3]. Trastuzumab exerts several biological effects, including anti-proliferation, apoptosis activities, antibody-dependent cellular cytotoxicity and phagocytosis against HER2-positive cells, and therefore inhibits tumour growth [4, 5]. In a previous study, Sorace et al. [6] employed DCE-MRI to identify and quantify vascular changes early in the course of treatment with trastuzumab in a murine model of HER2 + breast cancer. They found that trastuzumab-treated tumours exhibited a significantly increased perfusion and vessel maturation index on day four compared to controls. This finding suggests properties of vessel maturation, which is similar to “vascular normalization” induced by anti-VEGF therapies. Recent studies have proven that HER2 is an ideal target for treating these patients with trastuzumab [7, 8]. HLX02 (Zercepac® Henlius, Inc.) is an anti-HER2 trastuzumab biosimilar that was developed for more cost-effectiveness and accessibility [9]. In vitro studies have demonstrated that HLX02 shares an identical amino acid sequence, structure and function with trastuzumab [10]. However, the therapeutic efficacy has not been further confirmed.

Tumour vasculature is the cornerstone of tumour growth, metastasis and invasion. Immature tumour vessels, which are characterized by incomplete basilar membranes, little pericyte coverage and endothelial cells, induce high extravascular leakage and low blood transport efficiency [11]. This will further lead to a hypoxic microenvironment, insufficient drug delivery and even epithelial–mesenchymal transition (EMT). As a result, tumour cells acquire metastatic potential by transforming their epithelial phenotypes into mesenchymal phenotypes and therefore increasing invasiveness [12]. Initially, anti-vascular endothelial growth factor (anti-VEGF) or VEGF receptor therapy, which targets abnormal tumour vessels, was mainly used to inhibit tumour angiogenesis. However, clinical studies indicated that the tumour response rate or overall survival was not significantly improved after antiangiogenic monotherapy [13]. This may have resulted from the aggravated hypoxia level and hypoxia-related treatment resistance. With further research, antiangiogenic therapy has also been found to repair the vascular structure and normalize function, which is called vascular normalization [14]. This phenomenon has been regarded as an important treatment strategy and manifested an ideal therapeutic efficacy in various advanced tumours when combined with chemotherapy [15, 16]. Endostar (YH-16) is a novel recombinant human endostatin expressed and purified in *Escherichia coli* [17]. It is a strong endogenous inhibitor of angiogenesis that downregulates the expression of VEGF and its receptors [18]. However, its antitumor mechanism regarding vascular normalization in gastric cancer has not been clearly illustrated in clinical studies due to a lack of noninvasive examinations.

Magnetic resonance imaging (MRI) has become increasingly important in current anticancer research as various advanced sequences have been developed. Dynamic contrast-enhanced MRI (DCE-MRI) is regarded as the gold standard in perfusion imaging using contrast agents. A previous study confirmed that DCE-MRI can effectively monitor the entire process of vascular normalization in colon cancer after bevacizumab treatment [19]. However, the potential adverse effects of contrast agents are difficult to ignore. Intravoxel incoherent motion diffusion-weighted imaging (IVIM-DWI) is an entirely noninvasive imaging sequence that can sensitively detect water molecule diffusion and tissue microperfusion without the need for exogenous contrast agents [20, 21]. It also provides several quantitative parameters and the feasibility of continuous surveillance for treatment responses in a short time. In this study, we evaluated the treatment efficacy of the trastuzumab biosimilar HLX02 combined with Endostar and explored the tumour invasiveness and the phenomenon of vascular normalization using IVIM-DWI in a gastric cancer xenograft model during treatment.

## Materials and methods

### Cell culture

The MKN-45 human gastric cancer cell lines were acquired from the American Type Culture Collection (ATCC, Manassas, Virginia). We cultured the cells in Roswell Park Memorial Institute (RPMI) 1640 medium supplemented with 10% foetal bovine serum at 37 ℃ in a 5–10% CO_2_ aseptic environment to maintain the physiological pH (7.2–7.4).

### Model establishment and grouping

The Laboratory Animal Ethics Committee of our institution approved this animal experiment. A total of 40 female BALB/c nude mice (aged 6–8 weeks, weighed 20–22 g) were obtained from Beijing Vital River Laboratory Animal Technology Corporation (Beijing, China) and kept in a specific pathogen-free environment. We subcutaneously administered 0.2 ml of MKN-45 tumour cells (2 × 10^6^/ml) into the right flank of a mouse to establish the gastric cancer model. The tumour volume was calculated as (length × width^2^) × 0.523 mm^3^. Each dimension was measured with a slide calliper. When the tumour volumes reached an average of 300 mm^3^, we selected 28 mice and randomly divided them into four groups. They were treated with 0.9% saline (Group 1), Endostar (5 mg/kg, Group 2), HLX02 (5 mg/kg, Group 3), or Endostar combined with HLX02 (Group 4) via intraperitoneal injection every three days.

### MRI scanning

We performed MRI scanning in a 3.0 T superconductor clinical MR system equipped with an eight-channel wrist coil (MR750, GE Healthcare, Milwaukee, WI, USA). The main imaging protocol included T1-weighted imaging (T1WI), T2WI and IVIM-DWI. The detailed parameters of T2WI included repetition time/echo time (TR/TE), 2500/78 ms; slice thickness, 2 mm; slice spacing, 2 mm; field of view (FOV), 50 × 50 mm^2^; and matrix size, 384 × 288. The detailed parameters of T1WI included repetition time/echo time TR/TE, 400/11.5 ms; slice thickness, 2 mm; slice spacing, 2 mm; FOV, 50 × 50 mm^2^; and matrix size, 384 × 224. A single-shot, echo-planar imaging pulse sequence with a chemical shift-selective saturation technique was used to perform IVIM-DWI imaging. The detailed parameters of IVIM-DWI included TR/TE, 3000/102.4 ms; slice thickness, 3 mm; slice spacing, 3 mm; FOV, 70 × 56 mm^2^ and matrix size, 128 × 64. The diffusion gradients were applied in three orthogonal directions with 11 b values (0, 20, 50, 100, 150, 200, 400, 600, 800, 1200, 1600 s/mm^2^). All mice were intraperitoneally anaesthetized with 0.1% pentobarbital solution and fixed in the supine position before examination. MR scanning was performed before and on Days 5, 10, 15 and 20 after treatment.

### IVIM-DWI quantization

We performed the image analysis on an integrated postprocessing workstation (AW4.5, GE Healthcare, USA). Three parameters were extracted using the Functool-MADC software. The multiple b-values biexponential model employs a segmented fitting method. B < 200 mm^2^/s is regarded as a low b-value, which mainly reflects pseudodiffusion. The data in this range were fitted to the biexponential model to obtain perfusion values (D* and f) with the following equation: SI/SI_0_ = (1 – *f*) × exp(–*bD*) + *f* × exp(–*bD* ∗), where SI_0_ represents the mean signal intensity at b = 0, and SI is the signal intensity at other *b*-values. A monoexponential model is used to obtain the D value when the b-value is higher than 200 mm^2^/s due to the negligible influence of the D* and f values in this range. The equation is simplified as SI/SI_0_ = exp(–bD). The *B*-value is defined as the diffusion sensitivity coefficient. The D-value stands for the true diffusion coefficient, reflecting the microscopic motion of water molecules. The D* value represents the pseudodiffusion coefficient, reflecting microcirculation perfusion. The *F value* indicates the perfusion fraction, reflecting the percentage of microcirculation perfusion among the total diffusion effect [22]. We manually plotted the regions of interest (ROIs) on the largest tumour section referencing T2WI.

### Histological analysis

After the last MR scanning, all the mice were sacrificed for pathological examination, including haematoxylin and eosin (HE) staining, HER2, E-cadherin, TUNEL immunohistochemical staining, and α-smooth muscle actin (SMA) and CD31 immunofluorescent double-staining. The tumour was resected from each mouse, fixed with 4% paraformaldehyde, embedded in paraffin, and cut into 3-mm thick slices. E-cadherin is a marker of epithelial cells and predictor of epithelial–mesenchymal transition. All antibodies were provided by Servicebio Technology Co., Ltd. (Wuhan, China), and we performed histological staining according to the manufacturer**’**s instructions. α-SMA is generally expressed in mature vessels and is used to evaluate vascularization normalization by the ratio of positive α-SMA area to CD31 area, namely, the vessel maturity index (VMI). All the stained slices were observed under high magnification (× 200) using an Olympus BX 53 microscope. We detected the integrated optical density (IOD) and calculated the percentage of targeted cells stained by the abovementioned antibodies using Image-Pro Plus 6.0 software (Media Cybernetics, USA).

### Statistical analysis

The statistical analysis was performed using SPSS 21.0 software (IBM Corporation, USA) and plotted with GraphPad Prism 7.0 (GraphPad Software Inc., USA). The Kolmogorov–Smirnov test was performed to determine the data distribution type. Numerical results with Gaussian distribution were presented as the mean value with standard deviation (SD). One-way analysis of variance (ANOVA) was used to determine the difference between groups, and Student–Newman–Keuls q test was regarded as the post hoc test. The Pearson correlation test was used to determine the correlation strength between IVIM-DWI parameters and pathological indices. *P* < 0.05 was considered as significantly different.

## Results

### Treatment efficacy

**Fig. 1:**
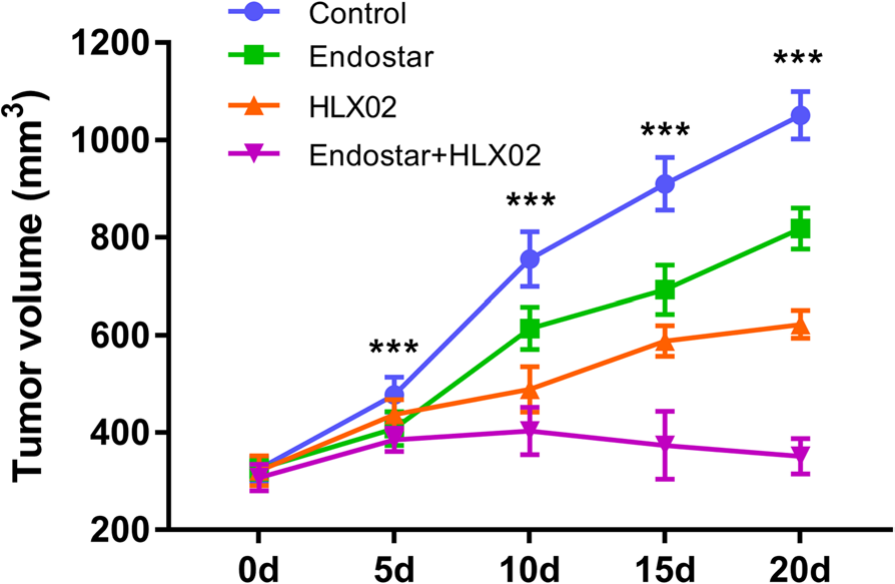
Tumour growth within 20 days in Groups 1, 2, 3 and 4. Seven rats were measured and averaged in each group. The smallest tumour volume with the highest tumour inhibition rate was observed in Group 4 at the end of treatment, followed by Groups 3, 2, and 1. *P < 0.05, **P < 0.01, and ***P < 0.001 were generated from comparisons between the four groups at each time point using one-way analysis of variance

All the mice stably endured the treatment courses with no obvious adverse reactions, such as vomiting and diarrhoea, or death. The mice in the three treated groups manifested smaller tumour volumes than those in the control group. In addition, the combination group showed the lowest tumour volumes and greatest tumour inhibition rate (66.6%), followed by Group 3 (40.8%) and Group 2 (22.2%) at the end of treatment. Compared with Endostar or HLX02 monotherapy, the tumour volume in Group 4 further decreased by 57% or 43%, respectively. The detailed tumour volumes of the four groups are shown in Fig. 1 and Table 1.

**Table 1:** The detailed tumor volumes of the four groups

Volume(mm^3^)	Base	5d	10d	15d	20d	F	P
Group 1	325.6 ± 25.3	477.7 ± 35.8	755.9 ± 56.3	910.3 ± 54.3	1051.1 ± 48.7	302.4	0.001
Group 2	325.6 ± 21.3	408.1 ± 34.5	614.0 ± 43.2	693.0 ± 50.6	818.0 ± 42.1	183.7	0.001
Group 3	321.4 ± 30.7	437.6 ± 29.9	489.0 ± 46.8	588.4 ± 31.3	622.4 ± 28.3	88.1	0.001
Group 4	307.9 ± 27.2	385.4 ± 23.9	403.1 ± 48.8	373.9 ± 69.6	351.4 ± 36.6	4.783	0.004

The *P*-value was generated by one-way analysis of variance

### MRI evaluation

**Fig. 2:**
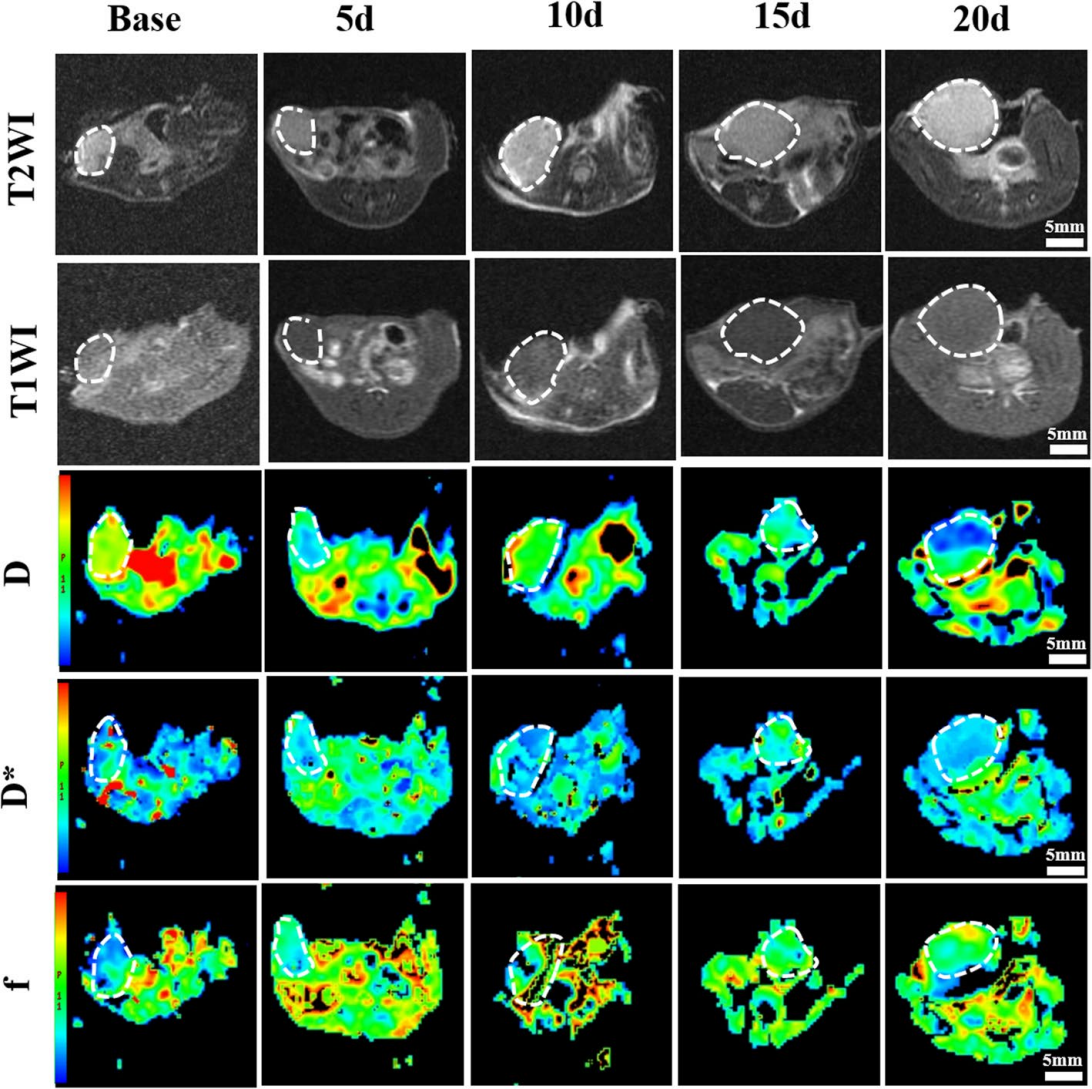
A representative group of T1WI, T2WI and pseudocolour maps of D, D ∗ , and f values (Group 4). The tumour areas are indicated by the white dashed circles. D, pure water molecule diffusion; D*, microcirculation perfusion; f, perfusion fraction

**Fig. 3:**
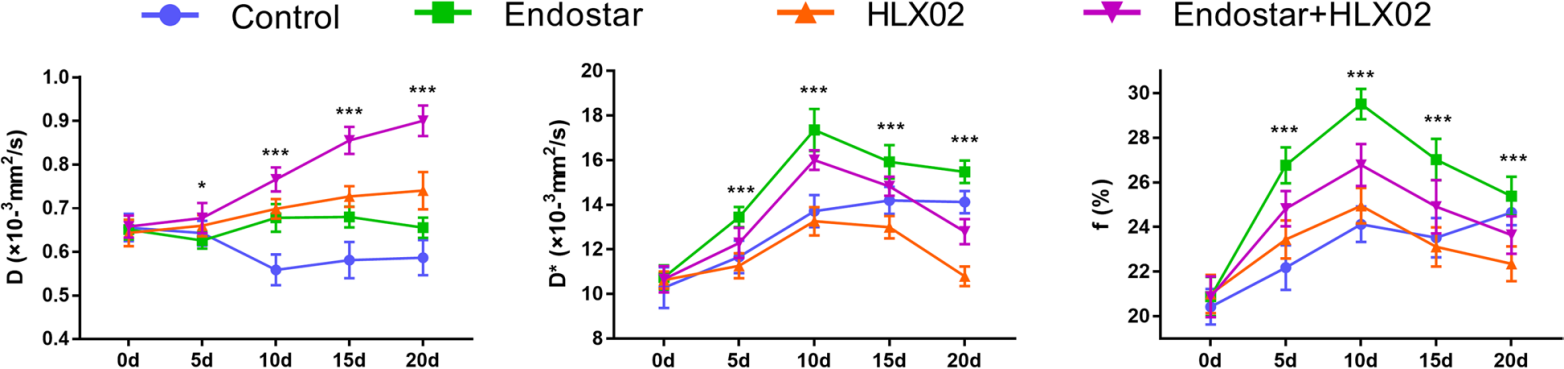
The dynamic changes in D, D ∗ , and f values in the treatment and control groups. Each data point represents the mean ± standard deviation. D, pure water molecule diffusion; D*, microcirculation perfusion; f, perfusion fraction. *P < 0.05, **P < 0.01, and ***P < 0.001 were generated from comparisons between the four groups at each time point using one-way analysis of variance

All mice were longitudinally monitored by IVIM-DWI to evaluate the microenvironmental changes during treatment. A representative group of T1WI, T2WI and pseudocolour maps of D, D ∗ , and f values are shown in Fig. 2. The detailed data of the D, D ∗ , and f values are listed in Table 2, and the trends are plotted in Fig. 3.

**Table 2:** The quantitative IVIM-DWI parameters at different time points from the four groups

Parameter	Groups	Base	5d	10d	15d	20d	F	P
D (10^−3^mm^2^/s)	Group 1	0.655 ± 0.031	0.643 ± 0.028	0.558 ± 0.035	0.581 ± 0.041	0.586 ± s0.040	9.705	0.001
	Group 2	0.650 ± 0.022	0.626 ± 0.018	0.678 ± 0.032	0.680 ± 0.024	0.655 ± 0.023	5.768	0.001
	Group 3	0.643 ± 0.030	0.660 ± 0.024	0.698 ± 0.023	0.727 ± 0.024	0.740 ± 0.043	13.717	0.001
	Group 4	0.658 ± 0.025	0.677 ± 0.035	0.766 ± 0.028	0.855 ± 0.031	0.900 ± 0.035	83.484	0.001
D* (10^−3^mm^2^/s)	Group 1	10.3 ± 0.9	11.7 ± 0.7	13.7 ± 0.7	14.2 ± 0.6	14.1 ± 0.5	42.501	0.001
	Group 2	10.7 ± 0.5	13.4 ± 0.5	17.4 ± 0.9	15.9 ± 0.8	15.5 ± 0.5	104.402	0.001
	Group 3	10.6 ± 0.4	11.3 ± 0.6	13.3 ± 0.6	13.0 ± 0.5	10.8 ± 0.4	42.051	0.001
	Group 4	10.7 ± 0.6	12.3 ± 0.7	16.0 ± 0.4	14.8 ± 0.4	12.8 ± 0.6	105.389	0.001
f (%)	Group 1	20.4 ± 0.8	22.2 ± 1.0	23.6 ± 0.8	23.0 ± 0.9	24.7 ± 0.6	26.388	0.001
	Group 2	20.9 ± 0.9	26.7 ± 0.8	29.5 ± 0.7	27.0 ± 0.9	25.4 ± 0.9	101.573	0.001
	Group 3	21.0 ± 0.9	23.4 ± 0.8	25.0 ± 0.8	23.1 ± 0.9	22.4 ± 0.8	21.451	0.001
	Group 4	20.9 ± 0.9	24.8 ± 0.8	26.8 ± 0.9	24.9 ± 1.2	23.6 ± 0.8	37.123	0.001

The *P*-value was generated by one-way analysis of variance

From the conventional T1WI and T2WI, the tumour size significantly increased all the time in the control group and was almost three to four times larger than the baseline value. In contrast, the tumour size increased much more slowly in Group 4 during treatment than in the other groups, indicating an ideal tumour inhibition effect under the combination treatment. However, we barely observed any signal changes, nor did we obtain quantitative data on T1WI and T2WI to characterize the microenvironmental changes during treatment.

For subsequent quantitative analysis of IVIM-DWI, the true diffusion coefficient-D value slowly decreased until Day 10 and was maintained at a low level afterwards in the control group (*F* = 9.705, *P* = 0.001), which may result from the increasing crowded cellularity due to tumour growth. In contrast, D values in Groups 2 (*F* = 5.768, *P* = 0.001) and 3 (*F* = 13.717, *P* = 0.001) moderately increased from Day 5 to Day 20 after treatment, while the trend was more dramatic in Group 4 (*F* = 83.484, *P* = 0.001). In addition, Group 4 exhibited the highest D value on Day 20. These results were interpreted as the extended extracellular space due to tumour necrosis under the effective treatments, especially when treated with HLX02 and Endostar.

For the perfusion-related parameters, the D* and f values in Group 1 gradually increased over time, which may be interpreted as the growing demand for blood supply and subsequent stimulation of tumour angiogenesis. D* (*F* = 42.051, *P* = 0.001) and f values (*F* = 21.451, *P* = 0.001) slightly increased until Day 10 and then decreased afterwards in the HLX02 group. The Endostar group and combination treatment group also demonstrated a similar increasing trend and peaked on Day 10. Group 2 showed the highest D* and f values on Day 10 compared with the other groups. Although the perfusion values started to decrease after Day 10 in Groups 2 and 4, the D* and f values between Day 5 and Day 20 were always higher than their baseline values, indicating increased tumour perfusion derived from vascular normalization. The downward trends of the tumour perfusion may result from the delayed anti-vascular effects as well as the recession of vascular normalization, prompting a transient time window after treatment with anti-angiogenic drugs.

### Pathological results

**Fig. 4:**
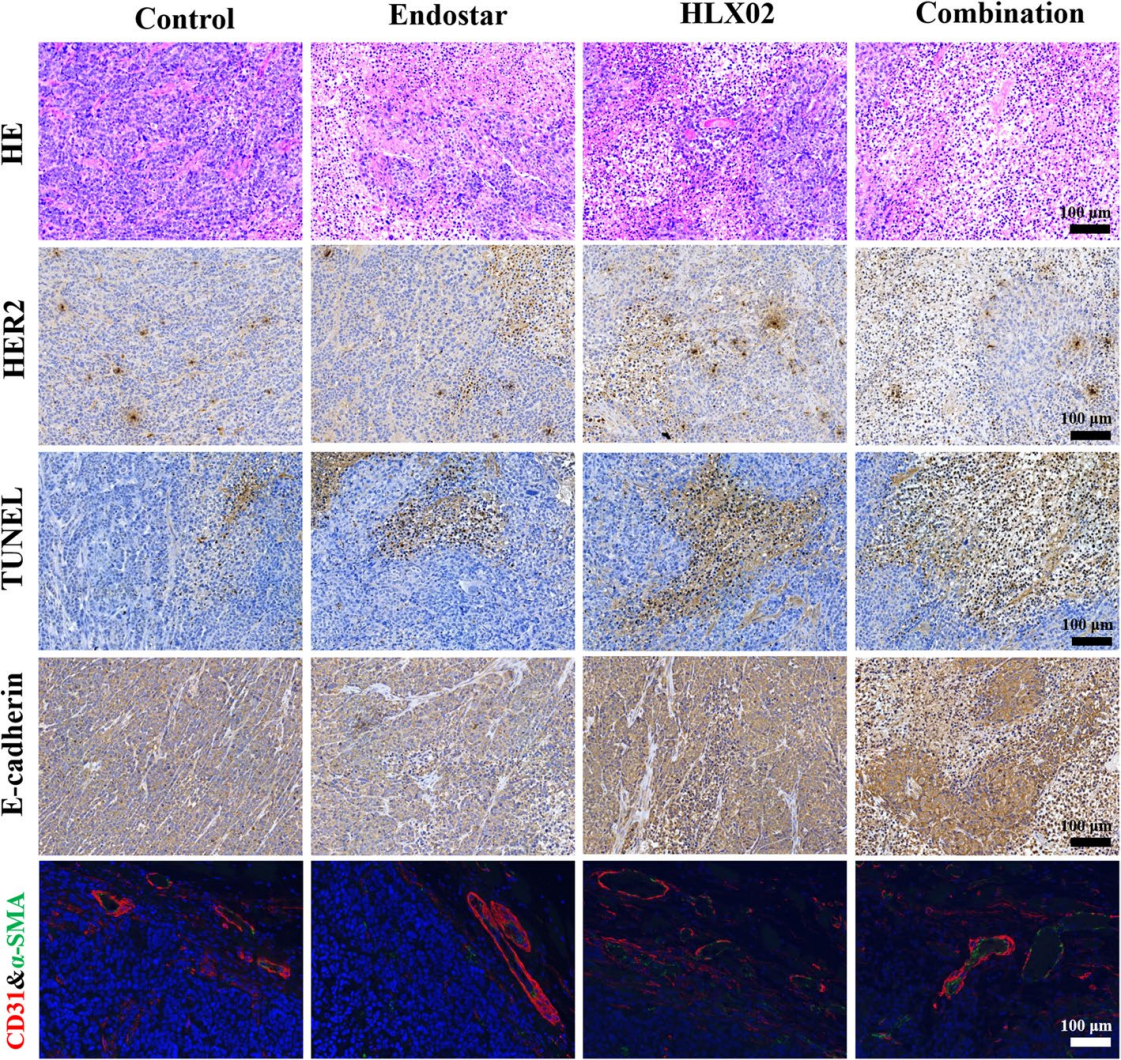
Representative sections of HE, HER2, TUNEL, E-cadherin and CD31 & α-SMA double staining in the four groups. HE, haematoxylin and eosin; HER2, human epidermal growth Factor 2; TUNEL, terminal-deoxynucleotidyl transferase mediated nick end labelling

**Fig. 5:**
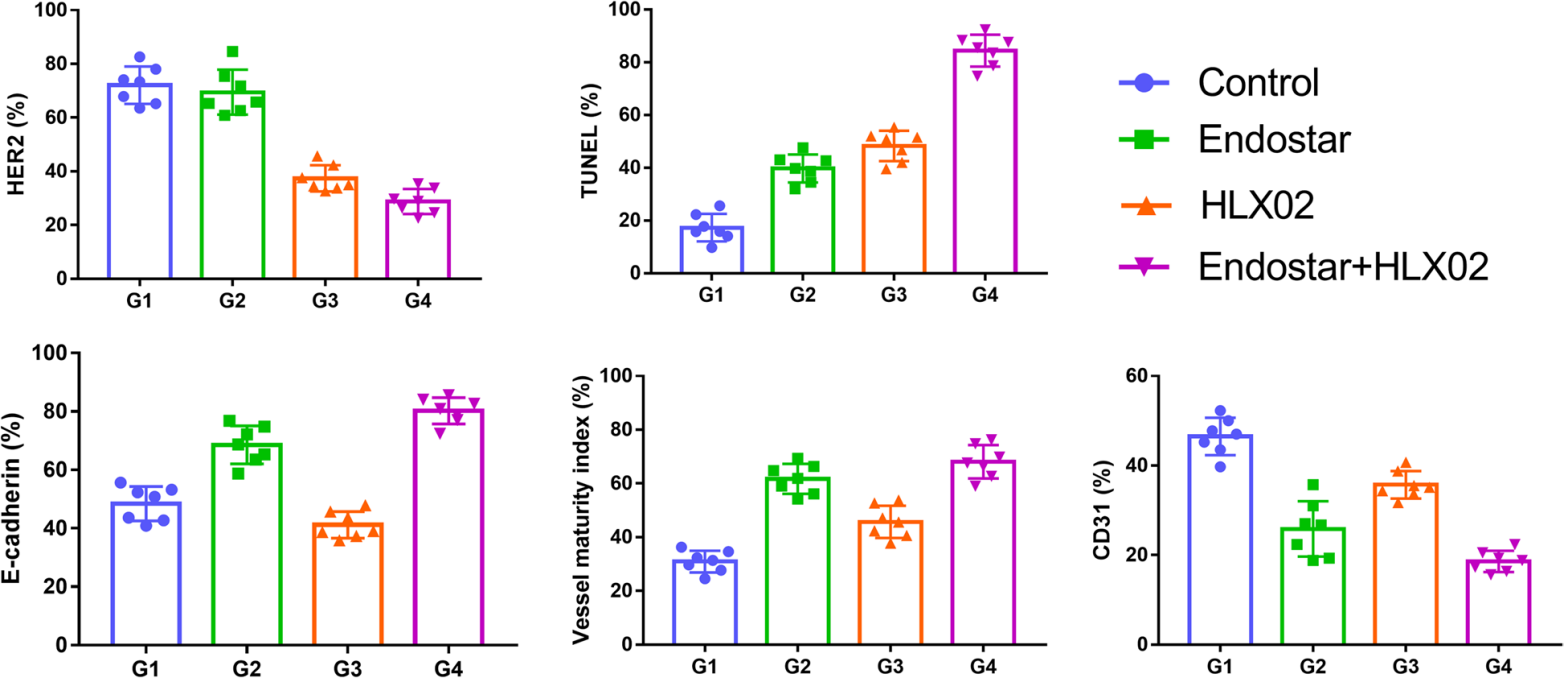
The bar charts of HER2, TUNEL, E-cadherin, VMI and CD31 quantifications in the four groups at the end of treatment. VMI, vessel maturity index; HER2, human epidermal growth Factor 2; TUNEL, terminal-deoxynucleotidyl transferase mediated nick end labelling

The mean values and SDs of CD31-, VMI-, HER2-, TUNEL- and E-cadherin-positive staining rates and their statistical results are listed in Table 3. Representative sections of HE, HER2, TUNEL, E-cadherin and CD31 & α-SMA double staining in the four groups are shown in Fig. 4. The bar charts of these results are shown in Fig. 5. For HE staining, the control group demonstrated much denser tumour cells with hyperchromatic nuclei in the section. Few necrotic areas were observed. However, the Endostar group showed a small patch of homogeneously red-stained area of necrosis in the tumour centre. Patches of haemorrhage and scattered necrosis were observed in the HLX02 group. Group 4 showed much larger areas of necrosis with more karyopyknosis and nuclear fragmentation in the section. HER2 staining showed decreased HER2 expression in Groups 3 and 4 compared with the control group (*F* = 83.141, *P* = 0.001). In addition, no significant difference was observed between Groups 1 and 2, suggesting that HLX02 can effectively target and inhibit the tumour cell proliferation activity induced by the HER2 receptor.

**Table 3:** The positive staining rates of CD31, VMI, HER2, TUNEL and E-cadherin in the four groups at the end of treatment

Index	Group 1	Group 2	Group 3	Group 4	F	P
CD31 (%)	46.5 ± 4.2	25.8 ± 6.2	35.7 ± 3.1	18.6 ± 2.4	57.962	0.001
VMI (%)	30.9 ± 4.0	61.7 ± 5.6	45.7 ± 6.0	68.1 ± 6.2	64.051	0.001
HER2 (%)	72.1 ± 7.0	69.5 ± 8.4	37.4 ± 4.9	28.8 ± 4.7	83.141	0.001
TUNEL (%)	17.3 ± 5.2	39.8 ± 5.3	48.3 ± 5.7	84.4 ± 6.0	174.221	0.001
E-cadherin (%)	48.4 ± 5.9	68.6 ± 6.5	41.2 ± 4.6	80.2 ± 4.5	76.733	0.001

The *P*-value was generated by one-way analysis of variance

*VMI* Vessel maturity index, *HER2* Human epidermal growth factor 2, *TUNEL* Terminal-deoxynucleoitidyl transferase mediated nick end labeling

For TUNEL staining, Groups 2 and 3 showed a moderate area of cell apoptosis in the tumour centre, but the efficacy of monotherapy was not quite satisfactory, as there still remained a large area of living cells, which may be the sources of recurrence. The combination group demonstrated a more radical treatment outcome, with cell apoptosis up to 90% at the end of treatment. The combination treatment induced another 44.6% or 36.1% tumour cell apoptosis compared with Endostar or HLX02 monotherapy.

E-cadherin staining showed increased expression of E-cadherin in Groups 2 and 4 compared with Groups 1 and 3, indicating a decreased metastatic potential due to vascular normalization and relieved tumour hypoxia. Regarding neovascularization, Group 4 exhibited a decrease of almost 60% of CD31-positive vessels and showed the lowest microvessel density at the end of treatment, followed by Groups 2 and 3, compared with the control group (*F* = 57.962, *P* = 0.001). The E-cadherin expression in Group 4 increased by approximately 40.7% or 8.6%. For vascular maturity, the VMIs were significantly improved in Groups 2 and 4, both of which were treated with Endostar. Interestingly, the VMI of Group 3 was also significantly higher than that of Group 1.

### Correlation results

**Fig. 6:**
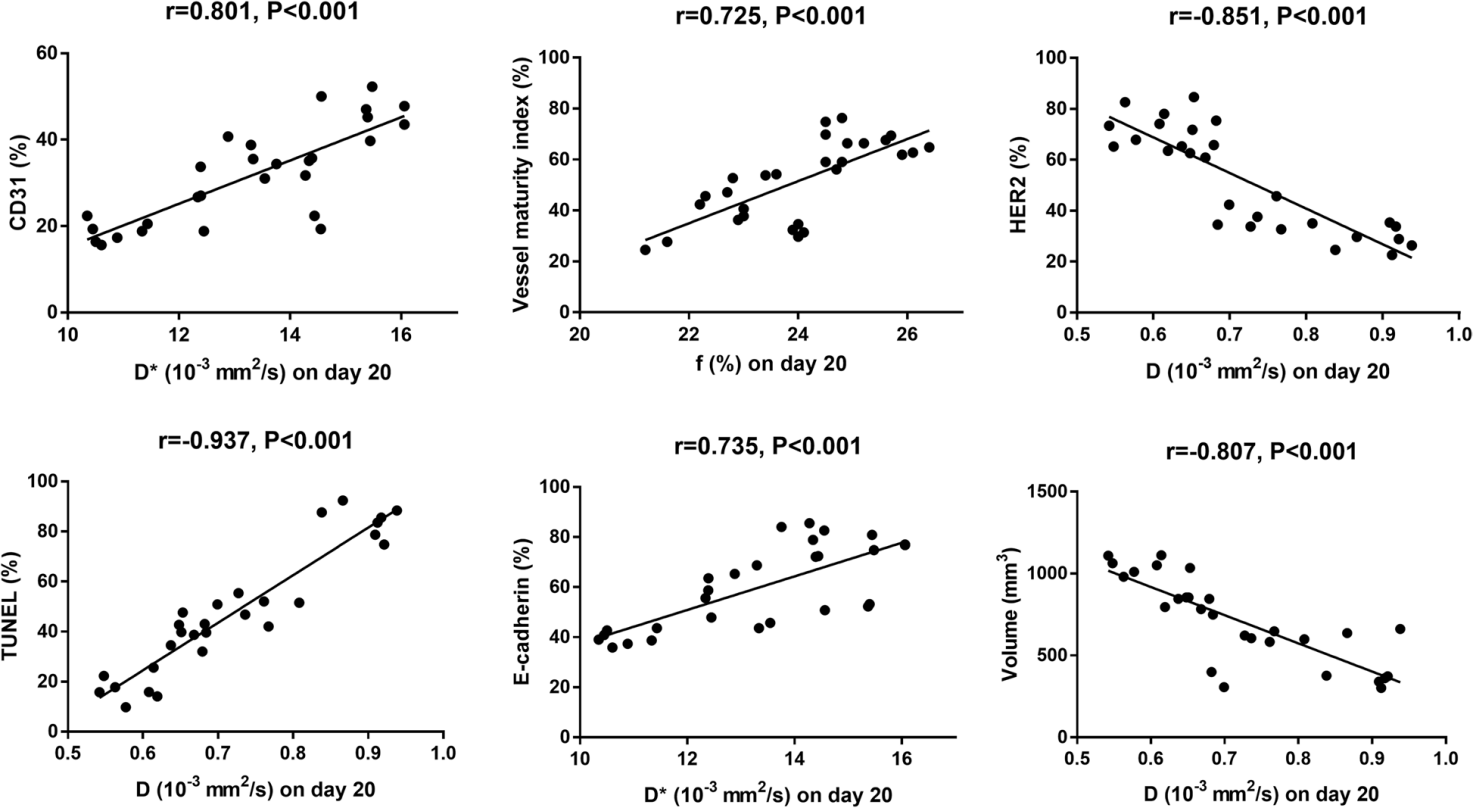
Scatter plots and fitted curves of the strongest correlations between pathological indices and MRI parameters. D, pure water molecule diffusion; D*, microcirculation perfusion; f, perfusion fraction; VMI, vessel maturity index; HER2, human epidermal growth Factor 2; TUNEL, terminal-deoxynucleotidyl transferase mediated nick end labelling

The correlation results between pathological indices and MRI parameters are shown in Table 4. The scatter plots and fitted curves of the strongest correlations between pathological indices and MRI parameters are shown in Fig. 6. When assessing tumour neovascularization, both D* (*r* = 0.801, *P* < 0.001) and f values (*r* = 0.776, *P* < 0.001) were highly and positively associated with microvessel density (CD31). In addition, D* (*r* = 0.801, *P* < 0.001) and f values (*r* = 0.776, *P* < 0.001) manifested relatively high correlations with VMI. Regarding tumour proliferation activity, the D value revealed a strong and negative correlation (*r* = -0.851, *P* < 0.001), while the D* (*r* = 0.589, *P* < 0.001) and f values (*r* = 0.579, *P* < 0.001) revealed moderate and positive correlations with HER2 expression. The D value demonstrated the strongest association with TUNEL staining (*r* = 0.937, *P* < 0.001) in evaluating tumour cell apoptosis, while the associations were weak in the D* (*r* = -0.384, *P* < 0.001) and f values (*r* = -0.393, *P* < 0.001). The D* value showed the strongest correlations with E-cadherin expression (*r* = 0.735, *P* < 0.001) in reflecting EMT and metastatic potential, followed by the f (*r* = 0.672, *P* < 0.001) and D (*r* = 0.524, *P* = 0.004) values. Finally, D values showed a strong and negative correlation with tumour volume in evaluating the overall efficacy (*r* = -0.807, *P* < 0.001).

**Table 4:** The relationships between pathological indexes and MRI parameters

Pearson coefficient	D		D*		f	
	r	P	r	P	r	P
CD31	0.237	0.224	**0.801**	0.001	0.776	0.001
VMI	0.376	0.048	0.706	0.001	**0.725**	0.001
HER2	**-0.851**	0.001	0.589	0.001	0.579	0.001
TUNEL	**0.937**	0.001	-0.384	0.044	-0.393	0.039
E-cadherin	0.524	0.004	**0.735**	0.001	0.672	0.001
Volume	**-0.807**	0.001	0.599	0.001	0.545	0.003

*VMI* Vessel maturity index, *HER2* Human epidermal growth factor 2, *TUNEL* Terminal-deoxynucleoitidyl transferase mediated nick end labeling

## Discussion

Currently, targeting the important oncogenic pathway based on molecular characteristics has become the nature of precision medicine and shows improved treatment outcomes in clinical practice. HER2 is a tyrosine kinase receptor and a well-established target in both breast and gastric cancers [23]. Previous studies have confirmed that HLX02 has comparable safety, pharmacokinetics and immunogenicity to European Union-sourced trastuzumab [24, 25]. In this study, we attempted to evaluate the efficacy of the trastuzumab biosimilar HLX02 combined with antiangiogenic treatment in a preclinical gastric cancer xenograft model. The microenvironment changes, including tumour cellularity, microcirculation perfusion, tumour invasiveness, and vascular normalization, were confirmed by MRI and pathological examinations.

In our study, the combination group manifested the smallest tumour volume, with the tumour inhibition rate reaching 66.6%, which was significantly higher than that of the monotherapy or control group. In addition, a moderate area of necrosis confirmed by HE staining and cell apoptosis confirmed by TUNEL staining were observed in the HLX02 group. Necrosis and apoptosis were significantly enhanced in the combination group. These results confirmed the effectiveness of HLX02 treatment in HER2-positive gastric cancer, especially when combined with antiangiogenic agents. We also performed IVIM-DWI and found that the D value had strong relationships with tumour volume (*r* = -0.807) and TUNEL staining (*r* = 0.937), suggesting that it can predict treatment efficacy early at the molecular level.

When comparing HER2 expression, we found that it was significantly decreased in Groups 3 and 4, which were treated with HLX02 and showed good efficacy. The results proved that HLX02 can inhibit HER2 expression and reduce cell proliferation activity during tumour growth. In the study of Sorace et al. [6], tumours treated with trastuzumab demonstrated significantly decreased Ki67 positive staining on Day 10 compared to baseline or the control tumour staining, revealing significantly decreased proliferation in treated tumours. In a previous study, Wang et al. explored the HER2 copy number of circulating tumour DNA in advanced gastric cancer patients treated with trastuzumab. They found that HER2 copy number decreased in most patients who benefited from trastuzumab but increased in those with progressive disease after treatment [26]. This result further supported our findings. Although Endostar did not directly target the HER2 marker, Group 4 still showed slightly lower HER2 expression than the HLX02 group. Both trastuzumab and Endostar can normalize the irregular tumour vasculature and provide a more efficient delivery of nutrients and drugs. The synergistic effect can further enhance the homogeneous spread of HLX02 across whole tumour tissues and effectively inhibit HER2 expression. We also found that the D value was negatively correlated with HER2 expression (*r* = -0.851), suggesting that it may serve as a prognostic surrogate for trastuzumab treatment in the future.

In our study, IVIM-DWI showed that perfusion obviously increased after 5 days not only in the Endostar group but also in the HLX02 group. The D* and f values in the treated group were always higher than the baseline values during Day 5 and Day 20 (P < 0.001), suggesting increased tumour perfusion due to vascular normalization. The results suggested that HLX02 targeting HER2 + cancers can also increase tumour perfusion and exert a synergistic effect on vascular normalization and cancer treatment in conjunction with anti-VEGF therapies. CD31 and α-SMA fluorescent staining confirmed the increased expression of α-SMA on the vascular wall with Endostar or HLX02 treatment. The results were similar to those of Liang et al., who confirmed that bevacizumab can also induce vascular normalization in a colon cancer model within a short time window between Days 3 and 12 after treatment [27]. Our study found that perfusion-related parameters showed high correlations with VMI. They can be used to predict the process of vascular normalization noninvasively.

Our study also found that E-cadherin expression was significantly increased in Groups 2 and 4, indicating decreased metastatic potential. E-cadherin, which tightly connects adjacent epithelial cells, is an indicator of EMT in a hypoxic microenvironment. The increased microcirculation perfusion and relieved tumour hypoxia would restore E-cadherin expression and reduce cell migration, which further relieves tumour invasiveness and increases drug delivery, indirectly enhancing the treatment efficacy.

There are several limitations in our study. First, we could not obtain tumour samples at other time points to confirm α-SMA expression and VMI. The correspondence between vascular maturity and perfusion parameters during the process of vascular normalization was unclear. Second, we did not perform hypoxia-related MRI or pathological examinations (such as BOLD-MRI and HIF-1α staining) in this experiment. Although increased perfusion was confirmed by MRI, there was no direct evidence to reveal a relieved hypoxic microenvironment. Third, only one gastric cancer cell line and subcutaneous model limited its general application for patients. We will compare the efficacy on more models with different gastric cancer cell lines and analyse orthotopic models in our future research. Finally, antiangiogenic and anti-HER2 treatments are generally used in some unresectable metastatic cancers with high expression of HER2. Their combination is also not a common therapeutic scheme in clinical practice, and chemotherapy should be incorporated for investigation in the future.

## Conclusions

The trastuzumab biosimilar HLX02 demonstrated a synergistic treatment efficacy when combined with Endostar. Both of them increased α-SMA expression and normalized vascular function in the gastric cancer model. The process of vascular normalization could be noninvasively reflected by the perfusion parameters of IVIM-DWI. Based on the high correlations of the D value with HER2 expression, cell apoptosis and tumour volume, the D value can be employed to predict the treatment efficacy and prognosis of HER2-positive cancer in the future.

## Data Availability

The datasets used during the current study are available from the corresponding author on reasonable request.

## Abbreviations

EMT: Epithelial–mesenchymal transition
VEGF: Vascular endothelial growth factor
MRI: Magnetic resonance imaging
IVIM-DWI: Intravoxel incoherent motion diffusion-weighted imaging; D, true diffusion coefficient
D*: Pseudo-diffusion coefficient
f: Perfusion fraction
VMI: Vessel maturity index
HER2: Human epidermal growth factor 2
TUNEL: Terminal-deoxynucleoitidyl transferase mediated nick end labeling
